# Cognitive Impairment and Its Associated Factors in Older Adults Living in High and Low Altitude Areas: A Comparative Study

**DOI:** 10.3389/fpsyt.2022.871414

**Published:** 2022-06-23

**Authors:** Shou Liu, Fei Wang, Cheng Zhang, Qinge Zhang, Zhan-Cui Dang, Chee H. Ng, Yu-Tao Xiang

**Affiliations:** ^1^Department of Public Health, Medical College, Qinghai University, Xining, China; ^2^Guangdong Provincial People's Hospital, Guangdong Mental Health Center, Guangdong Academy of Medical Sciences, Guangzhou, China; ^3^Department of Yong Ding lu Outpatient, Jingnan Medical Area, Chinese PLA General Hospital, Beijing, China; ^4^The National Clinical Research Center for Mental Disorders and Beijing Key Laboratory of Mental Disorders, Beijing Anding Hospital and The Advanced Innovation Center for Human Brain Protection, Capital Medical University, Beijing, China; ^5^Department of Psychiatry, The Melbourne Clinic and St Vincent's Hospital, University of Melbourne, Richmond, VIC, Australia; ^6^Unit of Psychiatry, Department of Public Health and Medicinal Administration, Institute of Translational Medicine, Faculty of Health Sciences, University of Macau, Macao, China; ^7^Centre for Cognitive and Brain Sciences, University of Macau, Macao, China; ^8^Institute of Advanced Studies in Humanities and Social Sciences, University of Macau, Macao, China

**Keywords:** cognitive impairment, older adults, high altitude, quality of life, comparative study

## Abstract

**Background:**

Cognitive impairment is a major health concern in older adults. Few studies have examined the association between environmental factors and cognitive impairment, especially in high altitude areas. In this study, the prevalence of cognitive impairment in older adults living in high altitude was compared with those living in low altitude areas.

**Methods:**

This was a comparative study conducted at Qinghai (high altitude group), and Guangzhou (low altitude group), China. Cognition, depressive symptoms and quality of life (QOL) were assessed using the Montreal Cognitive Assessment (MoCA), Patient Health Questionnaire (PHQ-9) and WHO Quality of Life brief version–WHOQOL-BREF, respectively.

**Results:**

Altogether, 644 older adults (207 in Qinghai and 437 in Guangzhou) completed the assessment. The prevalence rate of cognitive impairment was 94.7% (95% CI: 91.6–97.7%) in older adults living in the high altitude area, while the corresponding figure was 89.2% (95% CI: 86.3–92.1%) in the low altitude area. After controlling for covariates, the high altitude group appeared more likely to have cognitive impairment (OR = 2.92, 95% CI: 1.23–6.91, *P* = 0.015) compared with the low altitude group. Within the high altitude group sample, multinomial logistic regression analysis revealed that older age (aged 74 and above) was significantly associated with higher risk of severe cognitive impairment (OR = 3.58, 95%CI: 1.44–8.93, *P* = 0.006), while higher education level (secondary school and above) was associated with decreased risk of moderate cognitive impairment (OR = 0.43, 95%CI: 0.22–0.85, *P* = 0.006). Within the high altitude group, QOL did not differ significantly between normal/mild, moderate and severe cognitive impairment subgroups across physical [*F*_(1, 207)_ = 1.83, *P* = 0.163], psychological [*F*_(1, 207)_ = 1.50, *P* = 0.225], social [*F*_(1,207)_ = 2.22, *P* = 0.111] and environmental domains [*F*_(1,207)_ = 0.49, *P* = 0.614].

**Conclusion:**

This study found that cognitive impairment was more common among older adults living in the high altitude area. Regular screening and appropriate interventions should be provided to older adults in need.

## Introduction

Cognitive impairment refers to a person's cognitive state which presents as difficulties in remembering recent events or conversation, decline in memory or concentration, trouble with comprehension or expression, and deterioration of social skills (1). Cognitive impairment is a common health problem among older adults and a growing public health challenge due to population aging in many high- and middle-low income countries (2). A systematic review (3) found that the pooled prevalence of cognitive impairment in older Chinese population aged 60 years and above was 14.7%. Another community-based study (4) found that 30% of Chinese older adults aged over 80 years suffered from cognitive impairment. Cognitive impairment could result in negative consequences, including reduced quality of life (QOL), impaired functional outcomes and increased health care burden (5, 6).

The outcomes of cognitive impairment among older adults are diverse. Some people with cognitive impairment remain stable or even return to normal over time, while others develop severe neurological diseases such as dementia (1, 7, 8). A previous study found a conversion rate of transitioning from cognitive impairment to dementia was around 15% per year (9). Therefore, cognitive impairment could increase the risk of dementia (10), which is a major cause of disability among older adults. It was estimated that around 50 million persons suffered from dementia globally, and this figure is expected to increase to 152 million by 2050 (11), which presents as a major public health challenge (12). As such, early identification of cognitive impairment and understanding its contributing factors are important to reduce the risk and consequences of cognitive impairment in older adults.

The factors involved in cognitive impairment have been widely studied (13), and they include infections (14, 15), vitamin deficiency (16, 17), use of certain medications (e.g., oxybutynin and diphenhydramine and lorazepam) (18, 19), and certain major medical conditions such as stroke (20, 21) and brain injury (22, 23). However, the association between environmental factors, such as high altitude, and cognitive impairment in older adults, has been rarely reported.

Living in high altitude areas (e.g., ≥2000 m above sea level) for an extended period of time could increase the risk of chronic altitude sickness, heart disease and other related complications (24, 25) induced by lower blood oxygen level. Particularly, chronic hypoxemia could alter neurotransmitter function, which is strongly associated with general cognitive decline (26). Previous research found a relationship between high altitude exposure and impaired cognitive performance and negative mood effects (e.g., depression), most likely due to the low oxygen pressure (27–31). Therefore, it is reasonable to assume that living in high altitude areas may increase the risk of cognitive impairment.

To date, no studies have compared the pattern of cognitive impairment in older adults living in high altitude and low altitude areas. To reduce the negative outcomes of cognitive impairment, it is important to understand the potential effects of high altitude on cognitive impairment in older adults and its associated factors. Therefore, we compared the prevalence of cognitive impairment and its associated factors in older adults living in high-altitude with those living in low-altitude areas. As QOL is a widely used comprehensive health outcome in both clinical practice and research, we also examined the association between cognitive impairment and QOL in older adults living in high altitude area. Due to the potential negative effects of chronic hypoxemia on cognition (26), we hypothesized that cognitive impairment would be more common in older adults living in high altitude area compared to those living in low altitude area.

## Methods

### Study Design and Participants

This comparative study was conducted between September 1st and November 31st, 2019 in three public nursing homes in a high altitude area (Xining, Qinghai province; an average altitude of 2,300m), and one public nursing home in a low altitude area (Guangzhou, Guangdong province; an average altitude of 10 m). Due to the rapid economic expansion in China in recent decades, the traditional family structure in which younger adults and their parents and even grandparents live together in a common household has largely changed. Consequently, many older adults even those with low care needs, are placed in public nursing homes if they are deemed to need assistance with daily life. Older adults living in the participating nursing homes were consecutively invited to participate in the study during the study period. Inclusion criteria included: (1) 60 years or older; (2) the ability to comprehend the assessment. Exclusion criteria were: (1) dementia and intellectual disability, as determined by a review of health records. This survey was approved by the Ethical Committee of the University of Macau. All participants provided written informed consent.

### Data Collection

Basic sociodemographic and clinical characteristics were collected by a review of the health records in the nursing homes and also confirmed with a face-to-face interview by trained research assistants.

Cognition was measured using the Chinese version of the Montreal Cognitive Assessment (MoCA) (32, 33) that is a commonly used tool to assess global cognitive impairment in older adults (34, 35). The MoCA comprises 30 items, covering short-term memory, visuospatial abilities, executive function, attention, concentration, working memory, and language domains (32). The sum score ranges between 0 and 30, with a higher score indicating better cognitive function. A total score of 26 is used as the cutoff value to indicate the presence of cognitive impairment (36). Following other studies (37–39), the MoCA total score of ≤ 9 was considered “severe cognitive impairment”, 10–17 was considered “moderate cognitive impairment”, 18–25 was considered “mild cognitive impairment”, and the total score of ≥26 was considered “no cognitive impairment”.

QOL was measured using the validated Chinese version of the WHO Quality of Life brief version–WHOQOL-BREF (40, 41) that consists of 26 items covering physical, psychological, environmental, social domains, with a higher score indicating higher QOL. Depressive symptoms were measured using the self-reported validated Chinese version of the Patient Health Questionnaire (PHQ-9) (42, 43). The PHQ-9 consists of 9 items with each scored from 0 to 3. A higher score reflects more severe depressive symptoms.

### Data Analyses

Sociodemographic and clinical characteristics between the high and low altitude groups were compared using univariate analyses (e.g., chi-square test, two independent samples *t*-test and Mann-Whitney U-test). The independent association between high/low altitude groups and cognitive impairment (the MoCA total score of <26) was examined using binary logistic regression model, with cognitive impairment as the dependent variable, and high/low altitude area as independent variable after controlling for those that significantly differed in univariate analyses. In addition, sociodemographic and clinical characteristics between those with and without cognitive impairment among older adults living in high altitude area were conducted using univariate analyses. QOL between older adults with and without cognitive impairment living in high altitude area was compared using analysis of covariance (ANCOVA) after controlling for variables that significantly differed in the univariate analyses. Finally, demographic and clinical correlates that were independently associated with cognitive impairment among older adults living in high altitude area were examined by multinomial logistic regression analyses, with no or mild cognitive impairment as the reference group, and those with significant group difference as independent variables. Significance level was set as 0.05 (two-tailed).

## Results

In total, 657 older adults were invited to participate in the study; of them, 644 (207 in Qinghai and 437 in Guangzhou) fulfilled the study entry criteria and were assessed. The mean age of older adults was 78.1 ± 8.8 years in the high altitude area, and 81.5 ± 8.3 years in the low altitude area. The prevalence of cognitive impairment was 94.7% (95% CI: 91.6–97.7%) in the high altitude group; of which, 19.8% had mild cognitive impairment (95% CI: 14.4–25.2%), 50.7% had moderate cognitive impairment (95% CI: 43.9–57.5%), and 24.2% had severe cognitive impairment (95% CI: 18.3–30.0%). In contrast, the prevalence of cognitive impairment was 89.2% (95% CI: 86.3–92.1%) in low altitude group, of which 30.0% had mild cognitive impairment (95% CI: 25.7–34.3%), 28.2% had moderate cognitive impairment (95% CI: 23.9–32.4%), and 31.1% had severe cognitive impairment (95% CI: 26.8–35.5%).

In the whole sample, significant differences were found between the high and low altitude groups in age, ethnicity, education, perceived financial status, perceived health status, prevalence of cognitive impairment, number of major medical conditions and PHQ-9 total scores (Table 1). After controlling for these variables as covariates, the high altitude group were more likely to have cognitive impairment (OR = 2.92, 95%CI: 1.23–6.91, *P* = 0.015) compared to the low altitude group.

**Table 1:** Basic demographic and clinical characteristic of the whole sample.

	**The whole sample**		**Low altitude group**		**High altitude group**		**Statistics**		
	**(*****n*** **=** **644)**		**(*****n*** **=** **437)**		**(*****n*** **=** **207)**		
	***N***	**%**	***N***	**%**	***N***	**%**	**χ^2^**	***df*** **^a^**	***P***
Age group (years)							**11.46**	**1**	**0.001**
60–74	144	22.4	81	18.5	63	30.4			
≥75	500	77.6	356	81.5	144	69.6			
Male gender	252	39.1	160	36.6	92	44.4	3.62	1	0.057
Ethnicity (Han Chinese)	632	98.1	433	99.1	199	96.1	5.17	1	**0.023**
Married/cohabitating	136	21.1	87	19.9	49	23.7	1.19	1	0.275
Secondary school or above	274	42.6	198	45.3	76	36.7	4.24	1	**0.039**
Smoking	136	21.1	91	20.8	45	21.7	0.07	1	0.790
Perceived financial status							31.31	2	**<0.001**
Good	256	39.8	144	32.9	112	54.1			
Fair	290	45.0	210	48.0	80	38.6			
Poor	98	15.2	83	19.0	15	7.2			
Perceived health status							24.62	2	**<0.001**
Good	154	23.9	84	19.2	70	33.8			
Fair	374	58.1	282	64.5	92	44.4			
Poor	116	18.0	71	16.2	45	21.7			
Cognitive impairment	586	90.9	390	89.2	196	94.7	5.074	1	**0.024**
	**Mean**	**SD**	**Mean**	**SD**	**Mean**	**SD**	***T*** **/** ***Z***	***df^b^***	***P***
Number of major medical conditions*^d^*	3.2	1.7	3.5	1.6	2.55	1.7	7.27	642	**<0.001**
PHQ-9 total	3.0	3.9	1.6	2.8	6.02	4.1	−14.83	–^c^	**<0.001**

*df = degree of freedom; PHQ-9 = Patient Health Questionnaire-9*.

a *χ^2^ test*;

b *Two sample independent t-test*;

c *Mann-Whitney U test*;

b *Major medical conditions including included Hypertension, Cardiopathy, Stroke, Parkinson, Diabetes, Asthma, Chronic bronchitis Emphysema, Pulmonary disease, Liver disease, Nephropathy, Thyroid dysfunction, Arthritis, Cancer, racture/Osteoporosis/Humpback, Other diseases*.

*Bolded values: p < 0.05*.

Table 2 shows the comparison of sociodemographic variables and QOL of older adults living in high altitude between normal/mild, moderate and severe cognitive impairment sub-groups. Compared to those with normal/mild cognitive impairment, older adults with moderate and severe cognitive impairment were more likely to be older, and less likely to have higher education level. After controlling for age group and education level, QOL did not significantly differ across the physical [*F*_(1,207)_ = 1.83, *P* = 0.163], psychological [*F*_(1,207)_ = 1.50, *P* = 0.225], social [*F*_(1,207)_ = 2.22, *P* = 0.111] and environmental domains [*F*_(1,207)_ = 0.49, P = 0.614] between normal/mild, moderate and severe cognitive impairment sub-groups within the high altitude group.

**Table 2:** Socio-demographics of older adults with cognitive impairment living in high altitude area.

	**Mild or no**		**Moderate**		**Severe**		**Statistics**		
	**(*****n*** **= 52)**		**(*****n*** **= 105)**		**(*****n*** **= 50)**				
	***N***	***%***	***N***	***%***	***N***	***%***	**χ^2^**	***df*** **^a^**	***P***
Male gender	29	55.8	45	42.9	18	36.0	4.25	2	0.119
Age group (years)							8.37	2	**0.016**
60–74	23	44.2	31	29.5	9	18.0			
≥75	29	55.8	74	70.5	41	82.0			
Ethnicity (Han Chinese)	51	98.1	101	96.2	47	94.0	1.14	-^b^	0.579
Married/cohabitating	9	17.3	30	28.6	10	20.0	2.93	2	0.231
Secondary school or above	27	51.9	33	31.4	16	32.0	6.92	2	**0.031**
Smoking	11	21.2	27	25.7	7	14.0	2.75	2	0.253
Perceived financial status							3.75	-^b^	0.445
Good	27	51.9	62	59.1	23	46.0			
Fair	22	42.3	37	35.2	21	42.0			
Poor	3	5.8	6	5.7	6	12.0			
Perceived health status							5.71	-^b^	0.222
Good	23	44.2	36	34.3	11	22.0			
Fair	19	36.5	47	44.7	26	52.0			
Poor	10	19.2	22	21.0	13	26.0			
	**Mean**	**SD**	**Mean**	**SD**	**Mean**	**SD**	***F*** **/χ^2^**	***df^c^***	***P***
Number of major medical conditions	2.5	1.6	2.5	1.5	2.7	2.2	0.35	2	0.703
PHQ-9 total	6.0	4.5	6.6	3.9	7.0	3.8	4.96	–^d^	0.084
Physical QoL	13.6	2.4	13.2	2.1	12.8	1.8	2.0.36	2	0.097
Psychological QoL	12.9	2.4	13.5	2.0	13.3	2.3	1.06	2	0.348
Social QoL	13.1	2.5	13.6	2.0	13.3	1.8	1.89	2	0.153
Environmental QoL	13.0	2.4	13.3	2.1	13.2	1.7	0.46	2	0.635

*df, degree of freedom; PHQ-9, Patient Health Questionnaire-9; QoL, Quality of Life*.

a *χ^2^ test*;

b *Fisher's Exact test*;

c *Analysis of Variance*;

d *Kruskal-Wallis test*.

*Bolded values: p < 0.05*.

Table 3 shows the results of multinomial logistic regression analyses in high altitude group. Compared with those with normal/mild cognitive impairment group, older adults (aged 74 and above) were more likely to have severe cognitive impairment (OR = 3.58, 95%CI: 1.44–8.93, *P* = 0.006), while those with secondary school and above education level were less likely to have moderate cognitive impairment (OR = 0.43, 95%CI: 0.22-0.85, *P* = 0.006).

**Table 3:** Independent correlates of cognitive impairment in the high altitude group by multinomial logistic regression analysis.

	**Moderate vs Mild or no**			**Severe vs Mild or no**		
	***P***	**OR**	**95% CI**	***P***	**OR**	**95% CI**
Age group (≥75 years)	0.079	1.88	0.93-3.78	**0.006**	**3.58**	**1.44-8.93**
Education (secondary school or above)	**0.015**	**0.43**	**0.22-0.85**	0.051	0.44	0.19-1.00

*CI, Confidence Interval*.

*Bolded values: p < 0.05*.

## Discussion

This study found that the prevalence of cognitive impairment in older adults living in high altitude area was 94.7% (95% CI: 91.6–97.7%), which is significantly higher compared to those living in low altitude area. In this study cognitive impairment was common in older adults in high altitude area: the prevalence of mild cognitive impairment was 19.8%, moderate cognitive impairment was 50.7%, and severe cognitive impairment was 24.2%, while in low altitude area, the corresponding figure was 30.0%, 28.2%, and 31.1% respectively, which is significantly higher than previous findings. For example, the pooled prevalence of cognitive impairment was 14.71% among older Chinese population aged 60 years and above (3). A community-based study (4) found that 30% of Chinese older persons aged over 80 years experienced cognitive impairment. Cognitive impairment in older adults could be attributed to the natural brain dysfunction over time (13, 44, 45) or the consequence sequela of long-term chronic physical diseases such as hypertension (46), cardiovascular disease (47), and diabetes (48). Moreover, all participants were recruited from nursing homes in this study. Compared to their community-dwelling counterparts, older adults living in nursing homes in China usually have poor general health status with more severe and frequent physical problems including neurological diseases, which could partly explain the higher prevalence of cognitive impairment in this study.

We found that compared to the low-altitude group, older adults living in the high-altitude area were more likely to suffer from cognitive impairment with varying severity. This may be explained by the occurrence of hypoxia in high-altitude areas that could directly impair the function of central neural system, which could in turn worsen existing cognitive status (49–51). Additionally, cognitive impairment in older adults could be also related to the physical and/or psychiatric problems induced by hypoxia. For instance, sleep-disorder induced hypoxia is associated with increased risk for cognitive impairment (52). Similarly, previous studies (53–56) found that psychiatric problems (e.g., depression and anxiety) are associated with an increased risk of cognitive impairment in older population. In this study depressive symptoms measured by the PHQ-9 were more severe in older adults living in the high altitude area than those in the low altitude area, which could partly explain the higher prevalence of cognitive impairment in the high-altitude group. Multinomial logistic regression analysis found that in the high altitude group, those with higher education level were less likely to suffer from more severe cognitive impairment, which is consistent with previous findings in both younger (45, 57) and older adults (58). Previous studies also found that early life education improvement could lower the risk of cognitive impairment (59).

As a widely used health outcome measure, QOL is determined by the interactions between protective factors (e.g., better health status and social support) and risk factors (e.g., psychological and physical distress) (60, 61). Considering the adverse effects of cognitive impairment on daily life and social functioning, it is reasonable to assume that older adults with more severe cognitive impairment had a lower QOL. However, we did not find a significant difference of QOL between the different sub-groups severity of cognitive impairment in the high altitude group, which also contradicted previous findings (62, 63). This could be related to the inclusion of participants who were living in public nursing homes, in which the appropriate daily care and supports provided could have offset the negative impact of cognitive impairment on QOL.

Several limitations should be noted. First, this was a cross-sectional study, which limited the ability to infer causality between living in high altitude area and cognitive impairment. Second, for logistical reasons, demographic variables between the high and low altitude groups were not matched. However, the potential confounding effect of unmatched variables on the findings was controlled for using ANCOVA. Third, the sample size in the high altitude group was relatively small due to limited number of nursing homes in Qinghai province. Fourth, all participants were recruited from nursing homes using consecutive sampling, rather than from the community using random sampling. Thus the findings could not be generalized to older adults living in the community and need to be confirmed in these settings. Finally, some factors (e.g., presence, severity and treatment of certain neurological diseases) associated with cognitive impairment were not assessed due to logistical reasons. Furthermore, this study was only conducted at a population level, hence future quantitative and experimental research should be conducted to examine their relationships at an individual level.

In conclusion, cognitive impairment was common among older adults, particularly those living in the high altitude area. Regular screening and appropriate intervention should be provided to older adults in need. Further large community-based samples studies and cohort studies should be conducted to understand the relationship between living in high altitude area and cognitive impairment.

## Data Availability Statement

There are stringent restrictions in making the research dataset of the clinical studies publicly available. Readers and all interested researchers may contact Dr. YT Xiang, xyutly@gmail.com, to apply for exemptions from the participating institutions if appropriate.

## Ethics Statement

The study protocol was approved by the Ethical Committee of the University of Macau. The patients/participants provided their written informed consent to participate in this study.

## Author Contributions

Y-TX: study design. SL, FW, CZ, QZ, and Z-CD: collection, analyses, and interpretation of data. SL and Y-TX: drafting of the manuscript. CN: critical revision of the manuscript. All authors: approval of the final version for publication.

## Funding

The study was supported by the National Science and Technology Major Project for investigational new drug (2018ZX09201-014), the Beijing Municipal Science and Technology Commission (Z181100001518005), the University of Macau (MYRG2019-00066-FHS), the Natural Science Foundation of Qinghai Province (2019-ZJ-906), and the Natural Science Foundation of Qinghai University Medical College (2019-kyy-2).

## Conflict of Interest

The authors declare that the research was conducted in the absence of any commercial or financial relationships that could be construed as a potential conflict of interest.

## Publisher's Note

All claims expressed in this article are solely those of the authors and do not necessarily represent those of their affiliated organizations, or those of the publisher, the editors and the reviewers. Any product that may be evaluated in this article, or claim that may be made by its manufacturer, is not guaranteed or endorsed by the publisher.
